# Educational interventions to enhance support for balancing work and treatment in inflammatory bowel disease patients

**DOI:** 10.1007/s00535-025-02248-6

**Published:** 2025-04-12

**Authors:** Nobuhiro Ueno, Aki Sakatani, Katsuyoshi Ando, Seisuke Saito, Kojiro Sugimura, Kazuyuki Tanaka, Shinya Serikawa, Chisato Ishikawa, Momotaro Muto, Yuhei Inaba, Kentaro Moriichi, Mikihiro Fujiya

**Affiliations:** 1 Department of General Medicine, Asahikawa Medical University Hospital, Midorigaoka-Higashi 2 - 1- 1- 1, Asahikawa, Hokkaido 078 - 8510 Japan; 2 Department of Gastroenterological Sciences, Asahikawa Medical University, Asahikawa, Hokkaido Japan; 3 Division of Gastroenterology, Department of Medicine, Asahikawa Medical University, Asahikawa, Hokkaido Japan; 4 Furano Hospital, Furano, Hokkaido Japan; 5 Nakashibetsu Town Hospital, Nakashibetsu, Hokkaido Japan; 6 Asahikawa Kosei General Hospital, Asahikawa, Hokkaido Japan; 7 Nayoro City General Hospital, Nayoro, Hokkaido Japan; 8 Asahikawa Red Cross Hospital, Asahikawa, Hokkaido Japan; 9 Engaru Kosei General Hospital, Engaru, Hokkaido Japan; 10 IBD Center, Asahikawa City Hospital, Asahikawa, Hokkaido Japan

**Keywords:** Support for balancing work and treatment, Employment, Medical education

## Abstract

**Background:**

Inflammatory bowel disease (IBD) significantly impacts employment and work productivity, necessitating support for balancing work and treatment (SBWT). While SBWT systems have been formalized in Japan, awareness among healthcare professionals remains low. This study aimed to evaluate the effectiveness of an educational program on SBWT for healthcare professionals in Hokkaido, Japan.

**Methods:**

A 2-year questionnaire-based study was conducted across eight medical facilities in Hokkaido, Japan, from November 2022 to November 2024. The educational program, comprising lecture-based and self-directed learning formats, addressed six key components of SBWT. Pre- and post-program surveys assessed changes in awareness, interest, and behaviors related to SBWT.

**Results:**

Pre-program awareness of SBWT was low (36.7% among doctors, 28.2% among medical staff). Post-program, awareness increased significantly to 81.3% and 58.3%, respectively (*p* < 0.01). Interest in SBWT improved across several categories for both groups, with greater gains among medical staff. Behavioral changes, such as detailed employment-related consultations with IBD patients and improved reporting practices from medical staff to doctors, were observed but not statistically significant. Lecture-based learning was more effective than self-directed methods, in increasing awareness, interest, and engagement with SBWT, particularly for medical staff.

**Conclusions:**

The educational program successfully enhanced awareness and interest in SBWT, with lecture-based methods proving more effective for medical staff. These findings emphasize the need for tailored educational strategies based on baseline knowledge. Future initiatives should focus on sustaining knowledge acquisition, expanding programs nationwide, and assessing long-term impacts on healthcare practices and patient outcomes.

**Supplementary Information:**

The online version contains supplementary material available at 10.1007/s00535-025-02248-6.

## Introduction

Inflammatory bowel disease (IBD), encompassing Ulcerative colitis (UC) and Crohn’s disease (CD), is defined as a chronic inflammatory condition affecting the intestinal tract [1, 2]. IBD predominantly affects young individuals, and since no curative treatments are currently available, lifelong management is required. Effective support must also address the patient’s lifestyle and social circumstances. Various initiatives have been implemented to support IBD patients, but given that the disease often affects younger individuals, employment support has emerged as a significant issue. IBD is known to significantly impact employment rates and work productivity. Several studies have demonstrated that individuals with IBD face higher unemployment rates [3, 4], increased sick leave [5, 6], and greater work-related disability compared to the general population [7, 8]. Collectively, these studies highlight the profound effect of IBD on employment and productivity, emphasizing the critical need for effective disease management and workplace accommodations.

“Support for balancing work and treatment” (SBWT) is defined as an initiative designed to assist individuals who are motivated and capable of working despite their illness. Its goal is to enable patients to continue working while receiving appropriate treatment, ensuring that employment does not hinder access to medical care or lead to discontinuation of their working lives due to treatment demands. In Japan, SBWT has been promoted as part of the government’s efforts to reform workstyle practices. By 2020, these measures had become increasingly pressing, with IBD and other designated intractable diseases added to the list of conditions eligible for support.

However, awareness of SBWT within clinical practice remains limited. There is an urgent need to increase knowledge and develop human resources through educational programs. Moreover, establishing effective SBWT requires the involvement of multiple healthcare professionals, including doctors and other medical staff. Therefore, we developed and conducted an educational program on SBWT for healthcare professionals in Hokkaido, Japan to enhance awareness, interest, and engagement with SBWT for IBD patients. The purpose of this study was to evaluate the effectiveness of the educational program in achieving these goals.

## Methods

### Study design and ethics

This questionnaire-based survey was conducted among healthcare professionals at eight medical facilities in Hokkaido, Japan, between November 2022 and November 2024. The participating institutions included Furano Hospital, Nakashibetsu Town Hospital, Asahikawa Kosei General Hospital, Nayoro City General Hospital, Asahikawa Red Cross Hospital, Engaru Kosei General Hospital, Asahikawa City Hospital, and Asahikawa Medical University Hospital. The research methodology was reviewed and approved by the ethics committees of Asahikawa Medical University (Approval number C22101) and each participating institution, in compliance with the principles of the Helsinki Declaration (1964 and subsequent revisions).

This project was conducted over 2 years and included the following phases: questionnaire development (3 months), first questionnaire survey (3 months), data analysis and educational program preparation (6 months), educational program implementation and second questionnaire survey (6 months), and final data analysis and manuscript preparation (6 months).

### Geographic and demographic overview in Hokkaido

Hokkaido, the northernmost island of Japan, covers approximately 83,424 square kilometers, accounting for about 22% of the country’s total land area. The island has a population of approximately 5.22 million, with around 2.6 million individuals employed and an unemployment rate of 3.2% (approximately 80,000 people). Sapporo, the largest city in Hokkaido, has a population of approximately 2 million, with most residents concentrated within the city. Asahikawa, the second-largest city in Hokkaido, has a population of around 330,000 and serves as the largest urban center in the northeastern region of the island. Surrounding Asahikawa, the cities of Nayoro, Engaru, Nakashibetsu, and Furano each have populations of approximately 30,000.

Asahikawa is a major commercial hub in Hokkaido, with a diverse economy encompassing agriculture, forestry, manufacturing, and commerce. The city hosts numerous commercial facilities and tourist attractions, and its employment trends closely follow those of the broader Hokkaido region. The northeastern region beyond Asahikawa is known for its strong primary industries, including agriculture, livestock farming, and fishing, with tourism also playing a significant economic role. Employment in this region is largely centered around agriculture and tourism-related industries.

### IBD-treatment in hokkaido and characteristics of the participating facilities

Hokkaido is home to approximately 10,000 IBD patients. The eight medical facilities participating in this study are responsible for IBD treatment across a vast area in northeastern Hokkaido, collectively managing approximately 1500 patients. Among them, Asahikawa Medical University Hospital, Asahikawa Kosei General Hospital, and Asahikawa City Hospital manage a particularly high volume of outpatient IBD cases, accommodating approximately 70% of whole IBD patients. These institutions serve as specialized IBD centers, offering advanced medical care, including endoscopic surgery and treatment for severe cases. In contrast, Asahikawa Red Cross Hospital, Nayoro City General Hospital, Nakashibetsu Town Hospital, Furano Hospital, and Engaru Kosei General Hospital are general hospitals with gastroenterology departments that manage a broad range of gastrointestinal disorders, including IBD. While these hospitals are not dedicated IBD specialty centers, they function as regional core hospitals, providing comprehensive gastrointestinal care. All eight facilities have multiple medical departments, including surgery, and operate as general hospitals with inpatient beds for gastroenterology patients.

### Questionnaire survey

Anonymous self-administered questionnaires were used in this study. The details of the questionnaire are provided in Table 1. Questions 1–1 to 2–6 were common to both pre- and post-education programm, while Question 3–1 was included only in the post-education programm. Responses were structured as yes/no, multiple-choice, 5-point Likert scales (1–5), or free descriptions. The first questionnaires were distributed to healthcare professionals at each participating facility and collected upon completion. The second questionnaire was administered after the educational program and collected within 1 month of its completion. All completed questionnaires were then forwarded to Asahikawa Medical University, where they were aggregated and analyzed.

**Table 1:** Details of the questionnaire

	Question	Answer 1 (Doctor)	Answer 2 (Doctor)	Answer 1 (Medical staff)	Answer 2 (Medical staff)
1–1	Do you know the term “work–life balance support”?	Yes or no	−	Yes or no	−
1–2	Do you know the “Guidelines” created by the Ministry of Health, Labor and Welfare?	Yes or no	−	Yes or no	−
1–3	Have you ever provided consultation to IBD patients about working?	Yes or no	Number of consultations per year	Yes or no	Number of consultations per year
1–4	Only for those who selected “yes” in 1–3 Details of consultation (multiple answers possible)	Type of work	−	Type of work	−
		Job description	−	Job description	−
		Hours worked	−	Hours worked	−
		Leave of absence	−	Leave of absence	−
		Changing jobs	−	Changing jobs	−
		Finding a job	−	Finding a job	−
		Other	−	Other	−
1–5	Only for those who selected “yes” in 1–3 Did you have any difficulty in responding to the consultation?	Yes or no	−	Yes or no	−
1–6	Only those who selected “yes” in 1–3 should answer What did you have difficulty with? (Multiple answers possible)	Outside the scope of labor	−	Outside the scope of labor	−
		I don’t know the answer	−	I don’t know the answer	−
		I don’t have any room to maneuver	−	I don’t have any room to maneuver	−
		Lack of information	−	Lack of information	−
		The support recipient is unknown	−	The support recipient is unknown	−
1–7	Only for those who selected “yes” in 1–3 Actual response (multiple answers possible)	I haven’t done anything (just talking)	−	I haven’t done anything (just talking)	−
		Preparation of the doctor’s opinion letter	−	Report to the doctor	−
		Creating a medical certificate	−	Request for a medical certificate	−
		Writing a letter to your workplace	−	Writing a letter to your workplace	−
		Call the workplace	−	Call the workplace	−
		Contact the company doctor	−	Contact the company doctor	−
		Contacting hospital office staff	−	Contacting hospital office staff	−
		Other	−	Other	−
1–8	Have you ever provided work–life balance support for patients other than those with IBD?	Yes or no	Disease name	Yes or no	Disease name
2–1	Do you think it is necessary to support work–life balance?	1–5	−	1–5	−
2–2	Have you become interested in support work–life balance?	1–5	−	1–5	−
2–3	Do you want to put support for work–life balance into practice?	1–5	−	1–5	−
2–4	Do you think the patient’s quality of life will improve?	1–5	−	1–5	−
2–5	Do you think it will improve patients’ awareness of their treatment?	1–5	−	1–5	−
2–6	Do you think it will improve patient outcomes?	1–5	−	1–5	−
3–1	Course format for educational projects	Lecture or self-training	−	Lecture or self-training	−

### Educational program on work–life balance support

The educational program for SBWT consisted of six components: (1) general overview of IBD; (2) clinical practice issues in IBD treatment [the social background and stress of IBD patients have a significant impact on their lives and quality of life (QOL)]; (3) importance of team-based medicine (a. Multidisciplinary collaboration, b. Regional medical cooperation); (4) employment issues for IBD patients and the necessity of SBWT; (5) practical methods for implementing SBWT for IBD patients; and (6) future strategies and measures. These contents were created based on past research papers, guidelines published by the Ministry of Health, Labor and Welfare, and issues extracted from the initial questionnaire survey. All content of the educational program remains the same regardless of job type or years of experience. This program content was also designed to be understandable to beginners who have little or no experience of IBD treatment and all six components could be completed within 1 h. Participants were able to choose their preferred learning method: direct face-to-face lectures, watching recorded video lectures, or self-directed training using educational booklets. Four face-to-face lecture-style sessions were held during the study period. In the final section of the program (6. Future strategies and measures), the importance of holding medical conferences at each facility to share IBD patient information related to employment was emphasized. Participants were also encouraged to thoroughly document the content of consultations with IBD patients for physicians. These two points were explicitly stated in the booklet and reinforced through face-to-face and recorded video lectures.

All of booklets, video production, lectures, and email correspondence are done entirely within our research group. The data for the booklets and recorded video lectures were stored online. After access rights were granted to research collaborators at each facility, the materials were distributed to participants in the educational program. For face-to-face lectures, a question-and-answer session followed each presentation, while E-Mail-support was available for those who chose the self-training option.

### Endpoints and definitions

The level of awareness is defined as the percentage of respondents who answered “yes” to Questions 1–1 and 1–2 of the questionnaire survey. The level of interest is defined as the average score for each of the six items in Questions 2–1 to 2–6. The level of behavioral change is defined as the percentage of respondents who answered “yes” to Questions 1–3 to 1–8. Healthcare professionals were divided into two categories: doctors and medical staff. The medical staff category covers nurses, pharmacists, nutritionist, medical social workers, medical office staff, etc. Those who chose direct face-to-face lectures and watching recorded video lectures were defined as the lecture-based methods group, and those who chose self-directed training using educational booklets were defined as the self-training group. The primary endpoints of the study were changes in awareness and interest in SBWT before and after education program among doctors and medical staff. Secondary endpoints included behavioral changes before and after education program, a comparison of the effectiveness of the lecture-based and self-training methods, and the factors influencing awareness and interest in SBWT.

### Statistical analyses

Numerical data were reported as medians [interquartile ranges (IQRs)] or number (%). Student’s *t* test or Fisher’s exact test was used for comparisons of numerical values obtained from the questionnaires. Logistic regression analysis was conducted to identify factors influencing recognition, while multiple regression analysis was used to assess factors affecting interest. A *p* value of less than 0.05 was considered statistically significant. Statistical analyses were performed using SPSS for Windows (SPSS Inc., Chicago, IL, USA).

## Results

### Survey participants

Prior to the educational program, 60 questionnaires were collected from doctors and 340 from medical staff. Following the program, 48 questionnaires were collected from doctors and 226 from medical staff. The demographic details of the survey participants are summarized in Table 2. No significant differences were observed in gender, age, years of experience, occupation, or specialty among the doctors and medical staff before and after the educational program. Among the doctors, 25 participants (52.1%) attended the lecture-based course, while 23 (47.9%) completed the self-training course. Among medical staff, 90 participants (40.4%) attended the lecture-based course, and 133 (59.6%) participated in the self-training course.

**Table 2:** Demographic details of the survey participants

		Before education project	After education project	*p* value
Doctor	*N*	60	48	
Gender, male	*N* (%)	32 (53.3)	38 (79.2)	0.43
Age, under 40 years	*N* (%)	39 (65.0)	33 (68.8)	0.68
Years of work experience	Median (IQR)	9 (4.8–18.3)	8 (5–16)	
Specialist				
Fellow of the Japanese Society of Internal Medicine	*N* (%)	11 (18.3)	9 (18.8)	0.96
Board Certified Gastroenterologist of The Japanese Society of Gastroenterology	*N* (%)	27 (45.0)	20 (41.7)	0.73
Board Certified Fellow of the Japan Gastroenterological Endoscopy Society	*N* (%)	27 (45.0)	24 (50.0)	0.52
Lecture	*N* (%)	−	25 (52.1)	
Self-training	*N* (%)	−	23 (47.9)	

		Before education project	After education project	*p* value
Medical staff	*N*	340	223	
Gender, male	*N* (%)	59 (17.4)	31 (13.9)	0.27
Age, under 40 years	*N* (%)	179 (52.6)	97 (43.5)	0.06
Years of work experience	Median (IQR)	9 (4–16)	10 (4–19)	
Nurse	*N* (%)	247 (72.6)	168 (75.3)	0.48
Hospital ward work	*N*	123	89	−
Outpatient work	*N*	97	65	−
Other	*N*	27	14	−
Pharmacist	*N* (%)	46 (13.5)	21 (9.4)	0.14
Nutritionist	*N* (%)	18 (5.3)	11 (4.9)	0.85
Medical social worker	*N* (%)	21 (6.2)	12 (5.4)	0.69
Other	*N* (%)	7 (2.1)	11 (4.9)	0.07
Lecture	*N* (%)	−	90 (40.4)	−
Self-training	*N* (%)	−	133 (59.6)	−

### Changes in awareness of SBWT

**Fig. 1:**
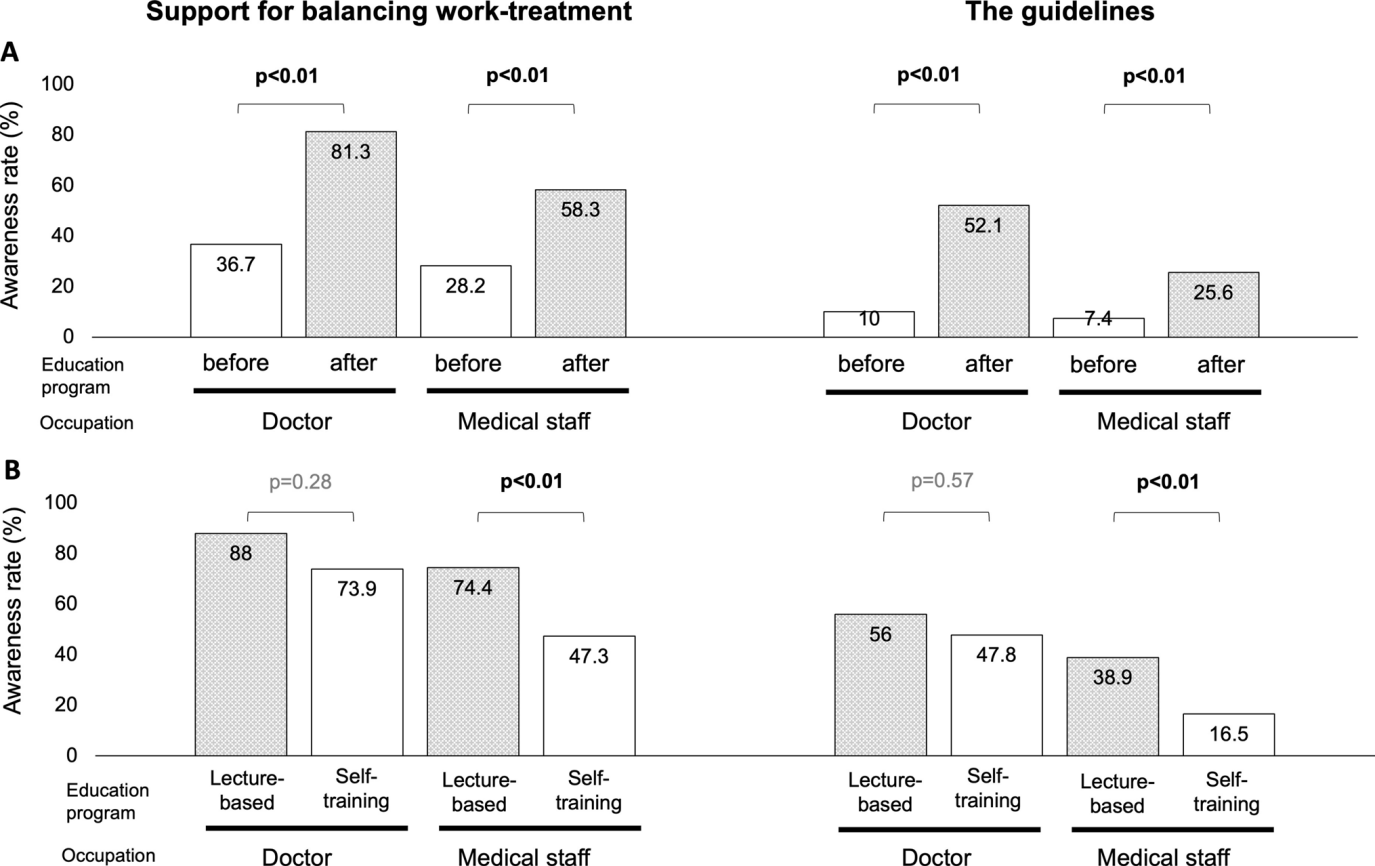
Changes in awareness rate of support for balancing work-treatment (SBWT) and guideline. **A** Before and after the educational program. **B** Between the lecture-based and self-training methods

The recognition survey was evaluated using a yes/no format, and the results are displayed in Fig. 1A. Before the educational program, awareness of SBWT was low, at 36.7% among doctors and 28.2% among medical staff. Awareness of the guidelines issued by Japan’s Ministry of Health, Labour and Welfare was even lower, at 10% among doctors and 7.4% among medical staff. After completing the educational program, awareness of SBWT increased significantly, rising to 81.3% among doctors and 58.3% among medical staff (*p* < 0.01 for both). Similarly, awareness of the guidelines increased significantly, to 52.1% among doctors and 25.6% among medical staff (*p* < 0.01 for both).

### Changes in interest in SBWT

**Fig. 2:**
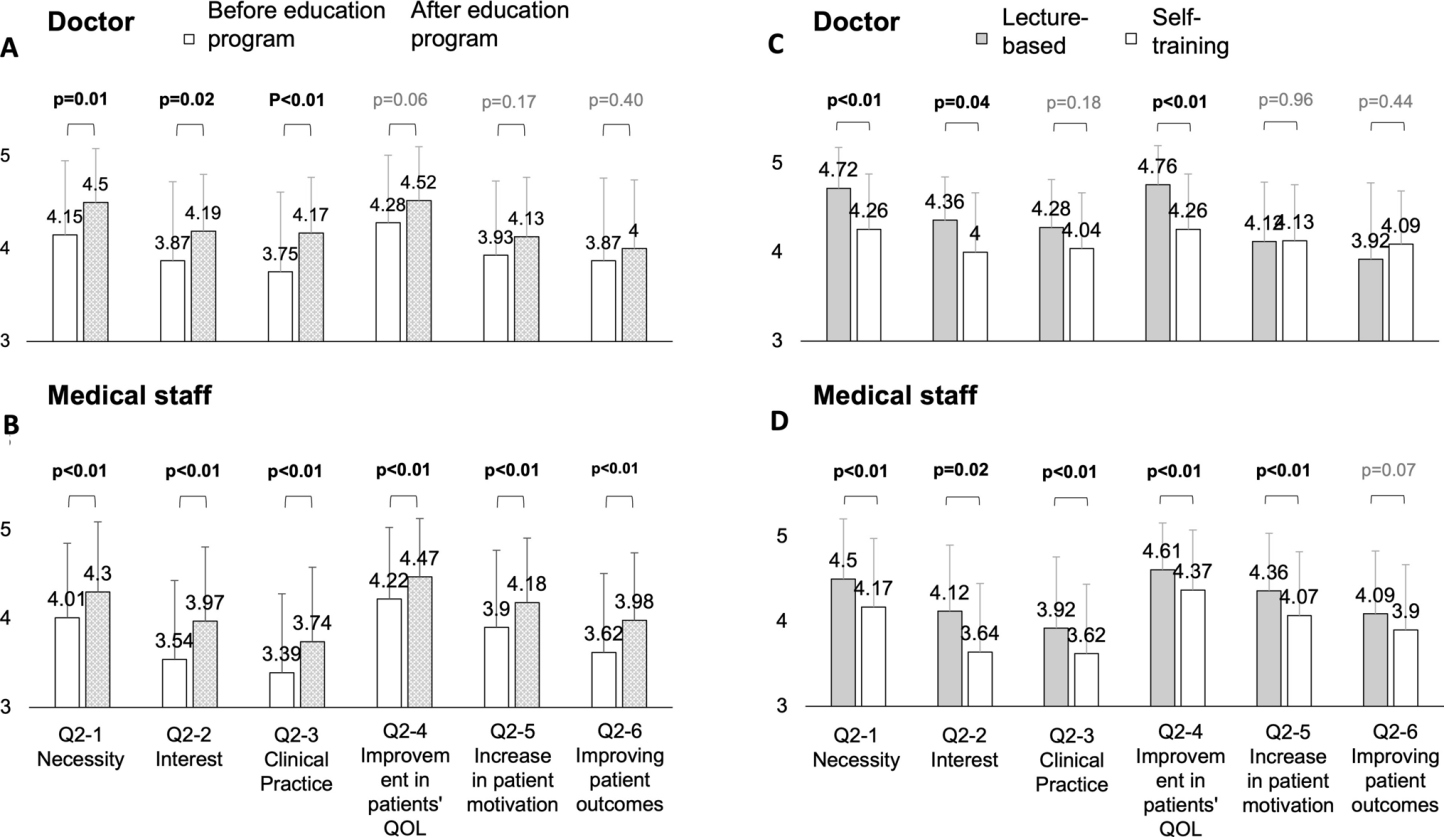
Changes in interest and concern about support for balancing work-treatment (SBWT). **A** Before and after the educational program among doctors. **B** Before and after the educational program among medical staff. **C** Between the lecture-based and self-training methods among doctors. **D** Between the lecture-based and self-training methods among medical staff

Participants were asked to rate their level of interest and concern regarding SBWT on a 5-point Likert scale. The results are presented in Fig. 2A, B. Both doctors and medical staff demonstrated increases across all six evaluated categories after completing the educational program. Among doctors, significant improvements were observed in the categories of “necessity” (4.15–4.5, *p* = 0.01), “interest” (3.87–4.19, *p* = 0.02), and “clinical practice” (3.75–4.17, *p* < 0.01). However, no significant changes were observed in the categories of “improvement in patients’ QOL” (4.28–4.52, *p* = 0.06), “increase in patient motivation” (3.93–4.13, *p* = 0.17), or “improving patient outcomes” (3.87–4, *p* = 0.40), which are directly relevant to IBD patients. In contrast, medical staff demonstrated significant increases across all six categories: “necessity” (4.01–4.3, *p* < 0.01), “interest” (3.54–3.97, *p* < 0.01), “clinical practice” (3.39–3.74, *p* < 0.01), “improvement in patients’ QOL” (4.22–4.47, *p* < 0.01), “increase in patient motivation” (3.9–4.18, *p* < 0.01), and “improving patient outcomes” (3.62–3.98, *p* < 0.01). Compared to doctors, medical staff perceived a greater impact of SBWT on factors directly related to IBD patients.

### Behavioral changes

**Fig. 3:**
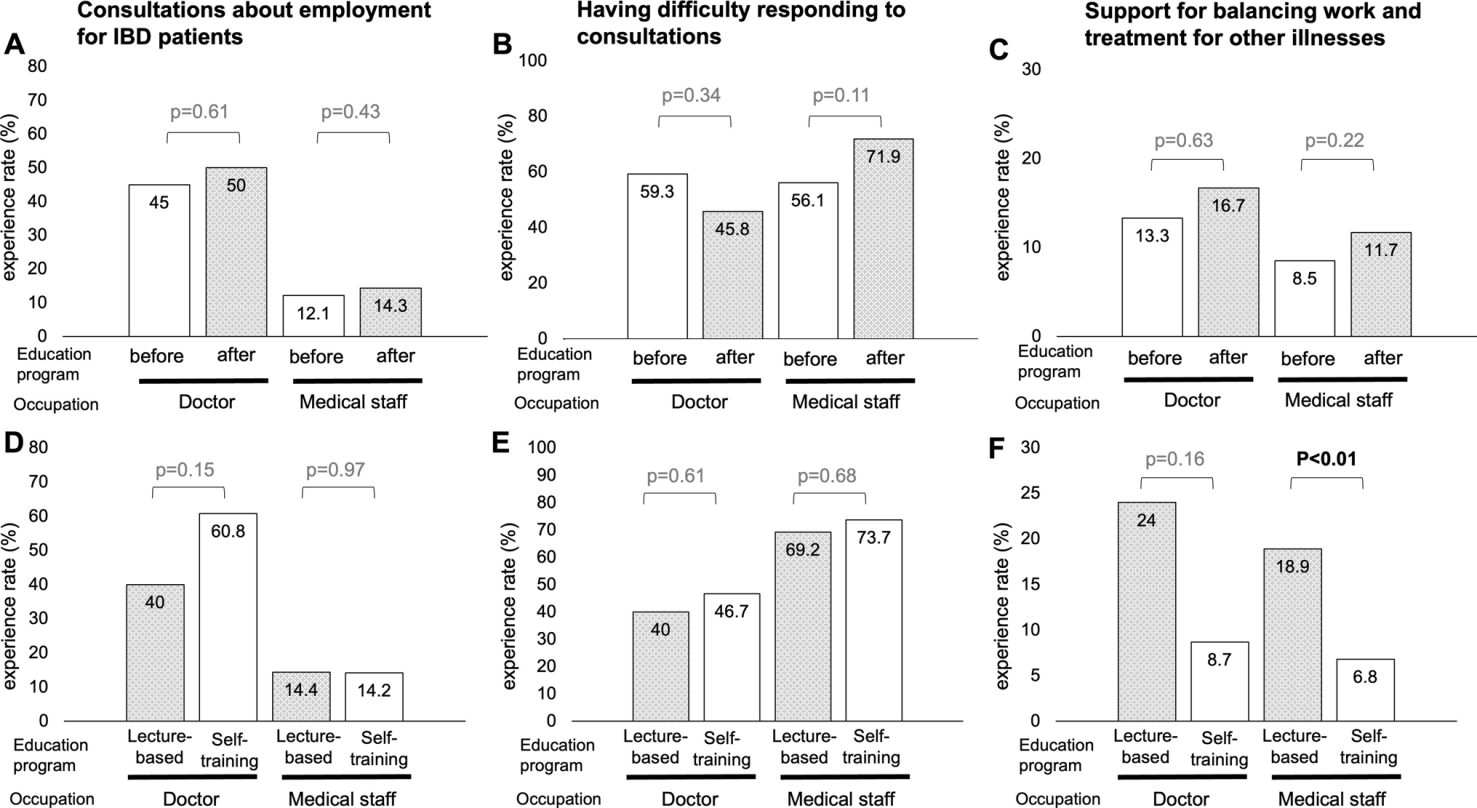
Behavioral change in support for balancing work-treatment (SBWT). **A**, **C**, **E** Before and after the educational program among doctors. **B**, **D**, **F** Before and after the educational program among medical staff

Behavioral changes resulting from the educational program were assessed in three areas: experience with consultations about employment for IBD patients, difficulty responding to such consultations, and experience providing SBWT for other illnesses. Responses were recorded using yes/no or multiple-choice formats, with results summarized in Fig. 3A–C. Before the educational program, 45% of doctors and 12.1% of medical staff had prior experience with consultations about employment for IBD patients. These findings indicate that doctors are the primary point of contact for employment-related consultations. After the program, the rate of such consultations increased slightly to 50% for doctors and 14.3% for medical staff, with no statistically significant difference compared to baseline (*p* = 0.61, *p* = 0.43, respectively). The percentage of respondents who reported difficulty in responding to consultations was high among both groups before the program, at 59.3% for doctors and 56.1% for medical staff. This proportion remained similarly high after the program, at 56.1% for doctors and 71.9% for medical staff, with no statistically significant decline (*p* = 0.34, *p* = 0.11, respectively). Experience in providing SBWT for illnesses other than IBD was limited before the program, at 13.3% for doctors and 8.5% for medical staff. These rates showed no significant increase after the program, rising to 16.7% for doctors and 11.7% for medical staff (*p* = 0.63, *p* = 0.22, respectively).

**Fig. 4:**
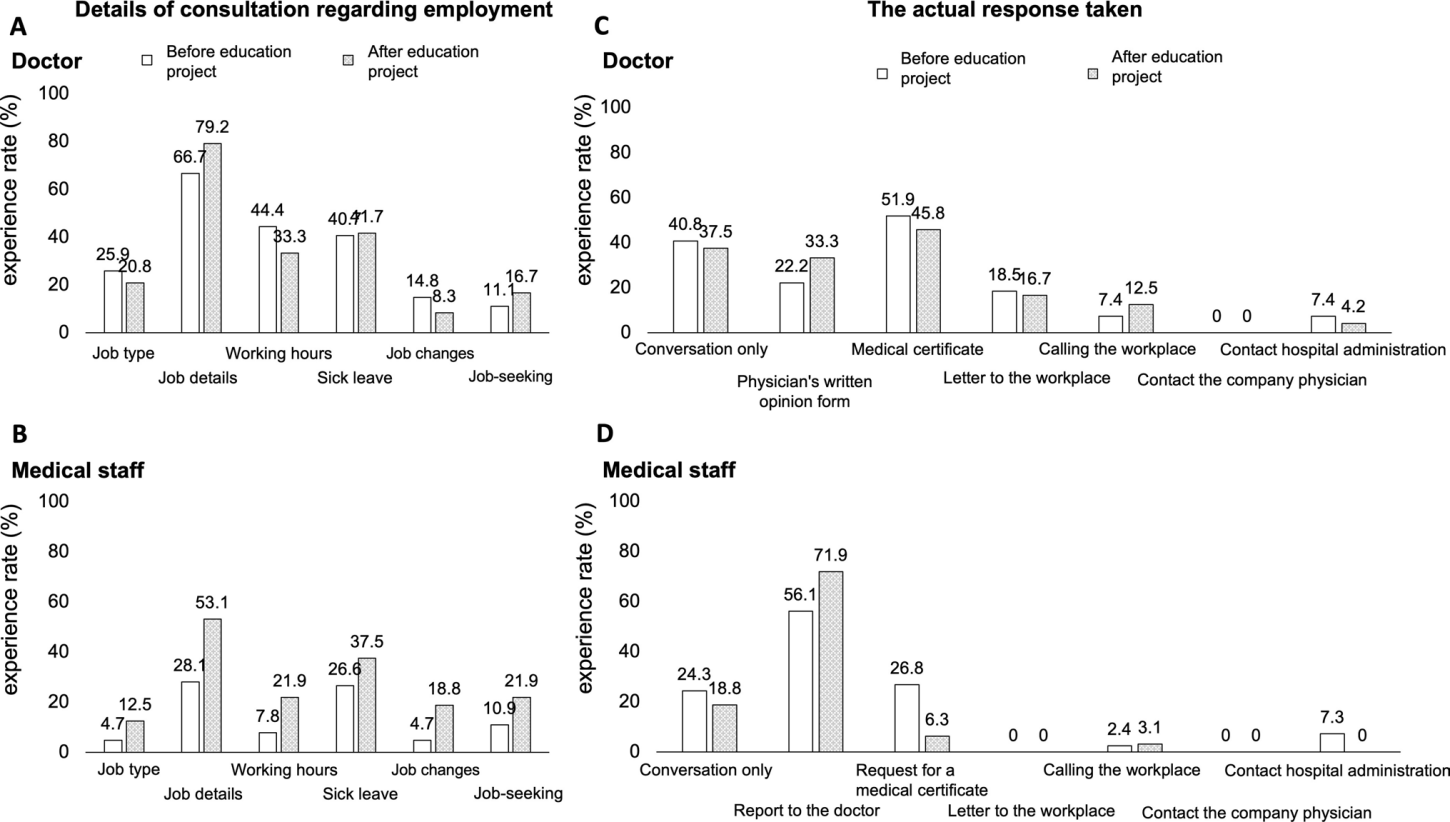
Changes in the detailed content of employment-related consultations and the actual responses taken. **A**, **C** Before and after the educational program among doctors. **B**, **D** Before and after the educational program among medical staff

The detailed content of employment-related consultations and the responses taken were further analyzed and are shown in Fig. 4A–D. Consultation content was categorized into six areas: job type, job details, working hours, sick leave, job changes, and job-seeking. Similarly, responses were classified into seven actions: conversation only, preparation of a physician’s written opinion, issuance of a medical certificate, letters to the workplace, direct calling with the workplace, contact with company physicians, and contact with hospital administrators. Among doctors, there was a tendency to more frequently inquire about the details of patients’ work, although no statistically significant increases were observed in specific categories. Among medical staff, the proportion of all consultation areas increased, with a similarly greater focus on job details. Regarding the types of responses provided by doctors, there was a notable trend of reduced reliance on medical certificates and increased use of physician-written opinions in alignment with the guidelines for SBWT. Among medical staff, there was a clear increase in reporting to doctors, reflecting a trend toward standardized responses. Although no significant behavioral changes were observed overall, the results suggest that the educational program initiated a process of unifying responses to employment-related consultations.

### Differences in effectiveness between lecture and self-training methods

As part of a sub-analysis, the effectiveness of the lecture-based and self-training methods was compared. The results are shown in Figs. 1B, 2C, D, and 3D–F. For doctors, there were no significant differences in the recognition of SBWT or the guidelines between the lecture-based and self-training methods (88% vs. 73.9%, *p* = 0.28; 56% vs. 47.8%, *p* = 0.57, respectively). However, the lecture-based method demonstrated significant improvements in “necessity” (4.72 vs. 4.26, *p* < 0.01), “interest” (4.36 vs. 4, *p* = 0.04), and “improvement in patients’ QOL” (4.76 vs. 4.26, *p* < 0.01). For medical staff, the lecture method was significantly more effective than self-training for both recognition of SBWT (74.4% vs. 47.3%, *p* < 0.01) and the guidelines (38.9% vs. 16.5%, *p* < 0.01). In addition, the lecture method resulted in significant improvements in “necessity” (4.5 vs. 4.17, *p* < 0.01), “interest” (4.12 vs. 3.64, *p* = 0.02), “clinical practice” (3.92 vs. 3.62, *p* < 0.01), “improvement in patients’ QOL” (4.61 vs. 4.37, *p* < 0.01), and “increase in patient motivation” (4.36 vs. 4.07, *p* < 0.01). In terms of behavioral changes, the experience rate of providing SBWT for other illnesses was significantly higher among medical staff in the lecture-based group compared to the self-training group (18.9% vs. 6.8%, *p* < 0.01).

These findings suggest that the lecture-based method was more effective overall, particularly for medical staff, in increasing awareness, interest, and engagement with SBWT.

### Factors influencing awareness and interest in SBWT

The factors influencing awareness and interest in SBWT were analyzed using multivariate analysis, considering both the overall results (before and after the educational program) and the results after the educational program alone. Interest in SBWT was assessed based on responses to the “2–2, Interest” section of the questionnaire. The overall analysis results are presented in Supplemental Table 3. Independent factors associated with awareness included participation in the educational program, age under 40 years, and more than 9 years of employment, with participation in the educational program exhibiting the highest odds ratio. Factors associated with interest included participation in the educational program, being a physician, and age under 40 years, with participation in the educational program also showing the highest coefficient, similar to awareness. The results of the analysis conducted after the educational program are presented in Supplemental Table 4. Independent factors associated with awareness included the lecture method, age under 40 years, and more than 9 years of employment, with the lecture method demonstrating the highest odds ratio. In addition, factors associated with interest included the lecture method and age under 40 years, with the highest coefficient observed for the lecture method, similar to awareness.

## Discussion

In this project, we developed and implemented an educational program on SBWT for IBD patients, targeting doctors and medical staff, and evaluated its effectiveness. As anticipated, baseline awareness of SBWT was very low. However, the educational program significantly improved awareness and increased interest in this support initiative, laying the groundwork for implementing standardized actions in clinical practice. Notably, the lecture-based method was more effective than the self-training approach, particularly for medical staff. This study represents the first report demonstrating the effectiveness of an educational program focused on SBWT for healthcare professionals treating IBD patients, and it provides valuable insights that may inform similar educational initiatives for other chronic illnesses.

IBD has a profound impact on patients’ employment and work productivity. The employment rate among Japanese IBD patients is 75.1%, lower than that of the general population [3]. Similar findings have been reported internationally, with higher unemployment rates among IBD patients [4]. Even for those who are employed, continuing to work can be challenging due to disease-related symptoms. A survey on the quality of working life for IBD patients found that symptoms negatively affect workplace performance and overall QOL [5]. The majority of IBD patients report difficulties at work, and unemployment is associated with lower QOL, as well as anxiety and depression [6]. Productivity losses due to absenteeism (absence from work) and presenteeism (productivity loss while at work) result in substantial economic costs. The WORK–IBD study reported that 18% of IBD patients experienced absenteeism, 50% experienced presenteeism, and 53% experienced overall work productivity loss, leading to mean annual indirect costs of €1738 for absenteeism, €5478 for presenteeism, and €6597 for overall work productivity loss [7]. A systematic review and meta-analysis revealed similar trends, with absenteeism rates averaging 16.4%, presenteeism at 35.9%, and overall work impairment at 39.4%, with associated annual indirect costs of €5,131.09 per patient [8]. Moreover, a Japanese study, The You and Ulcerative Colitis: Registry and Social Network (YOURS), found that even when UC symptoms were partially alleviated, patients continued to experience reduced work productivity [9]. This suggests that clinical response alone may not sufficiently improve work outcomes. Thus, while symptom control through intensified treatment is essential, providing appropriate work-support initiatives is equally important to enhance QOL and minimize societal losses.

In Japan, SBWT was formalized in 2016 through guidelines issued by the Ministry of Health, Labour and Welfare, and IBD was added to the list of target diseases in 2020. The process involves collaboration between patients, occupational health staff, and healthcare providers. First, the workplace’s occupational health staff prepares a “work information provision form,” which the patient submits to their attending physician. The physician then provides a “physician’s written opinion form,” which the patient submits to their workplace. This form is used as a reference to improve the working environment. However, this system relies on the initiative of IBD patients, many of whom may find it difficult to navigate these steps independently. Therefore, the active involvement of doctors and medical staff is critical. This requires healthcare professionals to first be adequately informed about SBWT. This study confirmed that awareness of both the support system and its associated guidelines was initially very low among healthcare professionals. In addition, the number of respondents to the second questionnaire survey was lower than that of the pre-program survey, particularly among medical staff. We suspect that the low level of awareness an interest in SBWT prior to the educational program may have influenced participants’ willingness to engage in the program. However, implementing an educational program aligned with the guideline content successfully raised awareness and interest. Despite these improvements, no significant changes in behavior were observed within the short interval between the educational program and the second questionnaire survey. Nevertheless, early indications of behavioral changes, such as a greater focus on understanding patients’ work details and standardizing reporting practices, were noted. Considering that 1 month elapsed between the survey and the completion of the educational program, the ability to observe even a small change can be considered a positive outcome. These findings suggest the need for a longer follow-up period to assess sustained behavioral change. Interestingly, the study also highlighted that doctors are the primary point of contact for IBD patients regarding employment issues. By improving awareness and interest among medical staff through our educational program, we hope to create an environment in which patients feel more comfortable consulting with a broader range of healthcare professionals about employment-related challenges.

This study also explored differences in the effectiveness of lecture-based and self-training methods. Lecture-based education is a traditional method for delivering structured information and is effective for imparting factual knowledge [10]. However, relying solely on lectures without interactive elements can hinder the development of practical skills and critical thinking [11]. Self-training methods, such as booklets, promote self-directed learning (SDL), a key component of lifelong learning in medicine. SDL encourages learners to identify their own needs, set goals, seek resources, and evaluate progress [12]. While SDL methods like e-learning offer flexibility and adaptability, they may not fully address areas requiring hands-on skills or interactive engagement [13, 14]. Previous several research reported that combining SDL with coaching or traditional lectures enhances the application of SDL strategies and improves satisfaction with clinical practice [15–18]. Our findings demonstrated that the lecture method was more effective in increasing interest among doctors, although no significant differences in awareness or behavioral change were observed. Among medical staff, however, the lecture method significantly outperformed self-training in improving awareness, interest, and behavioral outcomes. This difference may stem from the higher baseline experience of consultation about employment among doctors, making SDL more accessible to them, while medical staff, with less prior experience of consultation about employment, benefited more from structured lectures. These findings emphasize the importance of tailoring educational methods to the audience’s baseline knowledge. Combining traditional lectures with face-to-face coaching could be particularly effective for medical staff with limited opportunities to independently acquire new medical information. Although detailed data were not presented for questions posed after the educational program, no significant differences were observed between the lecture-based methods group and the self-training group. However, in the face-to-face lecture, actual case consultations occasionally took place during the question-and-answer session. The opportunity for direct discussion is considered a major advantage of the face-to-face approach.

Furthermore, this study examined the factors influencing awareness and interest in SBWT. The overall analysis, including both pre- and post-educational program assessments, demonstrated that the implementation of the educational program significantly influenced awareness and interest in SBWT. Furthermore, post-program analysis indicated that the educational method was the most influential factor, suggesting the effectiveness of the educational intervention. Additional factors were also identified as influencing awareness and interest in SBWT. Doctors exhibited a higher level of interest in SBWT compared to other medical staff. This may be attributed to their greater motivation to learn about SBWT, which could explain why the self-training method was as effective as the lecture-based method in increasing awareness. Moreover, individuals under 40 years of age demonstrated higher levels of awareness and interest in SBWT than those over 40, while those with more than 9 years of employment had greater awareness of SBWT than those with 8 or less years of employment. SBWT was established in 2016 and remains a relatively new system in Japan. Younger medical professionals may be more receptive to new initiatives and systems, such as SBWT. In addition, although IBD was incorporated into the SBWT guidelines in 2020, SBWT had already been implemented for other diseases prior to that. Thus, individuals with over 9 years of employment may have already been aware of SBWT in other medical fields before its application to IBD. Furthermore, longer medical professional experience naturally provides greater exposure to various clinical issues, increasing opportunities to recognize work-related challenges beyond IBD. However, the findings indicate that the number of employment years alone does not influence interest in SBWT, highlighting a critical issue. To enhance interest and encourage proactive implementation of SBWT, we believe that education itself plays a crucial role. However, this method of analysis has certain limitations. Since the questionnaire survey for this study was conducted anonymously, it was not possible to identify the individuals whose awareness and interest actually increased, introducing a significant source of bias. To enhance the educational program, it is essential to consider not only occupation but also age and years of experience. Further verification and evaluation will be necessary to address these factors.

There are also some other limitations to this study. It was conducted in the northeastern region of Hokkaido, Japan, a region characterized by primary industries and medium-sized cities, potentially limiting its generalizability to other regions with differing occupational health environments. In addition, the educational program was designed and delivered by a single instructor, which may introduce bias. As the venues for the face-to-face lectures varied, participants could attend only once. However, they could watch the recorded video lectures multiple times. In addition, participants had the option to engage in multiple learning methods, such as attending face-to-face lectures, watching recorded video lectures, and self-training using the booklets. Whether any participants watched the video lectures more than once or utilized multiple learning methods remains unclear, as this was not investigated. In addition, this study did not assess the amount of time participants actually spent on self-training using the booklet. Future studies should evaluate the program’s effectiveness across diverse settings, detailed learning status, and involve multiple educators to address these limitations. Finally, this study is looking at the overall 2-year process, and the results are based on a short-term effectiveness study. Continuous medical education (CME) is vital for healthcare professionals to maintain and enhance their competencies. Competency-based education (CBE) is an educational approach that aims to acquire specific skills, knowledge and abilities. CBE which emphasizes measurable outcomes over traditional time-based training, ensures that healthcare providers acquire skills aligned with evolving demands in patient care [19, 20]. E-learning offers an efficient and accessible means of delivering CME, particularly for time-constrained professionals [14]. Although this project demonstrated short-term effectiveness, sustainability remains a challenge. Having achieved a foundational level of knowledge through lecture-based programs, future efforts should incorporate SDL approaches, such as e-learning, transitioning to a CBE framework to encourage ongoing learning and measurable skill development. The ultimate goal of this initiative is to facilitate observable behavioral change among healthcare providers, leading to improved SBWT for IBD patients.

In conclusion, this educational project successfully increased awareness and interest in SBWT among healthcare professionals in Hokkaido, Japan. In particular, the lecture-based program proved highly effective for medical staff, demonstrating the importance of matching educational methods to the target audience’s baseline knowledge. As a foundational step toward establishing comprehensive SBWT systems for IBD patients, this initiative highlights the value of tailored education corresponds to the knowledge and experience of target group. Future efforts should focus on sustaining knowledge acquisition through continuous educational programs, expanding the initiative nationwide, and assessing its long-term impact on behavioral change and IBD patient outcomes.

## Supplementary Information

Below is the link to the electronic supplementary material.

Supplementary file1 (DOCX 34 KB)

Supplementary file2 (DOCX 34 KB)

## Abbreviations

IBD: Inflammatory bowel disease
UC: Ulcerative colitis
CD: Crohn’s disease
SBWT: Support for balancing work and treatment
IQRs: Interquartile ranges
QOL: Quality of life
SDL: Self-directed learning
CME: Continuous medical education
CBE: Competency-based education
