# Supplementary material for: Educational interventions to enhance support for balancing work and treatment in inflammatory bowel disease patients
Source: J Gastroenterol. 2025 Apr 12;60(8):967–78. doi: 10.1007/s00535-025-02248-6 (PMC12289813; doi:10.1007/s00535-025-02248-6)
Supplement: Supplementary file 1 — Supplementary file1 (DOCX 34 KB) [file 535_2025_2248_MOESM1_ESM.docx]

**Supplemental Table 3**

|  |  |  | Logistic regression analysis | | |  | Multiple regression analysis | | |
| --- | --- | --- | --- | --- | --- | --- | --- | --- | --- |
| Variable |  | Awareness, N (%) | p value | Odds ratio | 95% CI | Interest, average | p value | Coefficient | 95% CI |
| Education program | Pre | 118 (29.5) | <0.01 | 4.34 | 2.99 – 6.29 | 3.59 | <0.01 | 0.425 | 0.28 – 0.56 |
|  | Post | 169 (62.4) |  |  |  | 4.01 |  |  |  |
| Occupation | Doctor | 61 (56.5) | 0.09 | 0.62 | 0.36 – 1.09 | 4.01 | <0.01 | -0.32 | -0.53 – -0.11 |
|  | Medical staff | 226 (40.1) |  |  |  | 3.71 |  |  |  |
| Age | Under 40-year-old | 160 (45.9) | <0.01 | 0.47 | 0.31 – 0.71 | 3.87 | <0.01 | -0.32 | -0.40 – -0.10 |
|  | Over 40-year-old | 127 (39.4) |  |  |  | 3.64 |  |  |  |
| Gender | Male | 91 (50.8) | 0.77 | 0.93 | 0.58 – 1.51 | 3.78 | 0.08 | -0.25 | -0.02 – 0.34 |
|  | Female | 196 (39.8) |  |  |  | 3.75 |  |  |  |
| Employment years | 8 years or less | 99 (36.9) | 0.02 | 2.61 | 1.74 – 3.92 | 3.83 | 0.76 | 0.16 | -0.13 – 0.17 |
|  | More than 9 years | 154 (51.5) |  |  |  | 3.75 |  |  |  |
