# Supplementary material for: Educational interventions to enhance support for balancing work and treatment in inflammatory bowel disease patients
Source: J Gastroenterol. 2025 Apr 12;60(8):967–78. doi: 10.1007/s00535-025-02248-6 (PMC12289813; doi:10.1007/s00535-025-02248-6)
Supplement: Supplementary file 2 — Supplementary file2 (DOCX 34 KB) [file 535_2025_2248_MOESM2_ESM.docx]

**Supplemental Table 4**

|  |  |  | Logistic regression analysis | | |  | Multiple regression analysis | | |
| --- | --- | --- | --- | --- | --- | --- | --- | --- | --- |
| Variable |  | Awareness, N (%) | p value | Odds ratio | 95% CI | Interest, average | p value | Coefficient | 95% CI |
| Education methods | Self-training | 80 (51.3) | <0.01 | 3.21 | 1.72 – 5.97 | 3.88 | 0.02 | 0.26 | 0.02 – 0.48 |
|  | Lecture | 89 (77.4) |  |  |  | 4.17 |  |  |  |
| Occupation | Doctor | 39 (81.3) | 0.13 | 0.47 | 0.18 – 1.24 | 4.19 | 0.11 | -0.24 | -0.54 – -0.06 |
|  | Medical staff | 130 (58.3) |  |  |  | 3.97 |  |  |  |
| Age | Under 40-year-old | 89 (68.4) | 0.02 | 0.46 | 0.23 – 0.91 | 4.12 | 0.02 | -0.23 | -0.43 – -0.04 |
|  | Over 40-year-old | 80 (57.1) |  |  |  | 3.91 |  |  |  |
| Gender | Male | 53 (76.8) | 0.55 | 0.78 | 0.34 – 1.78 | 4.03 | 0.22 | 0.17 | -0.10 – 0.43 |
|  | Female | 116 (57.4) |  |  |  | 4.00 |  |  |  |
| Employment years | 8 years or less | 58 (55.2) | <0.01 | 3.16 | 1.61 – 6.23 | 4.00 | 0.09 | 0.11 | -0.03 – 0.40 |
|  | More than 9 years | 89 (71.8) |  |  |  | 4.07 |  |  |  |
